# Evolutionary Trend Analysis of Research on Immunotherapy for Brain Metastasis Based on Machine-Learning Scientometrics

**DOI:** 10.3390/ph17070850

**Published:** 2024-06-28

**Authors:** Xiaoqian Hu, Xinpei Deng, Jindong Xie, Hanqi Zhang, Huiting Zhang, Beibei Feng, Yutian Zou, Chuhuai Wang

**Affiliations:** 1 Department of Rehabilitation Medicine, The First Affiliated Hospital, Sun Yat-sen University, Guangzhou 510080, China; 2 School of Biomedical Sciences, Faculty of Medicine, The University of Hong Kong, Hong Kong, China; 3 State Key Laboratory of Oncology in South China, Guangdong Provincial Clinical Research Center for Cancer, Sun Yat-sen University Cancer Center, Guangzhou 510060, China; 4 Department of Rehabilitation Medicine, The Sixth Affiliated Hospital of Sun Yat-sen University, Guangzhou 510655, China

**Keywords:** cancer, immunotherapy, brain metastases, immune checkpoint inhibitor, bibliometric

## Abstract

Brain metastases challenge cancer treatments with poor prognoses, despite ongoing advancements. Immunotherapy effectively alleviates advanced cancer, exhibiting immense potential to revolutionize brain metastasis management. To identify research priorities that optimize immunotherapies for brain metastases, 2164 related publications were analyzed. Scientometric visualization via R software, VOSviewer, and CiteSpace showed the interrelationships among literature, institutions, authors, and topic areas of focus. The publication rate and citations have grown exponentially over the past decade, with the US, China, and Germany as the major contributors. The University of Texas MD Anderson Cancer Center ranked highest in publications, while Memorial Sloan Kettering Cancer Center was most cited. Clusters of keywords revealed six hotspots: ‘Immunology’, ‘Check Point Inhibitors’, ‘Lung Cancer’, ‘Immunotherapy’, ‘Melanoma’, ‘Breast Cancer’, and ‘Microenvironment’. Melanoma, the most studied primary tumor with brain metastases offers promising immunotherapy advancements with generalizability and adaptability to other cancers. Our results outline the holistic overview of immunotherapy research for brain metastases, which pinpoints the forefront in the field, and directs researchers toward critical inquiries for enhanced mechanistic insight and improved clinical outcomes. Moreover, governmental and funding agencies will benefit from assigning financial resources to entities and regions with the greatest potential for combating brain metastases through immunotherapy.

## 1. Introduction

Brain metastases pose a significant concern given their prevalence, which is expected to impact one-third of people with solid tumors [1], with the highest incidences being observed in melanoma (28%), lung cancer (26%), renal cell carcinoma (11%), and breast carcinoma (7.6%) [2]. Patients diagnosed with cancer and brain metastases are faced with a dismal prognosis, including increased morbidity and mortality [3], as well as a heavier financial burden [4]. The two and five survival rates for cancer patients suffering from different primary tumors with brain metastases are estimated to be 8.1 and 2.4%, correspondingly. Nonetheless, the cause of death in over half of these cases is attributed to the CNS metastases [5]. Whilst the combination of treatments has been conducive in ameliorating clinical outcomes [3], it often results in long-lasting adverse effects, such as hearing loss and neurocognitive impairment. These adverse events are predominantly observed in cases where patients receive chemotherapy and radiation therapy, regardless of the improvement in tumor-specific survival.

Notwithstanding, the therapeutic options available for patients with brain metastases primarily focus on alleviating symptoms rather than curing the disease. These options include brain-wide radiation therapy, stereotactic radiosurgery, and surgical resection, as well as combinations of these treatments [6]. Moreover, the clinical application of chemotherapy for brain metastases is constrained due to its suboptimal performance in traversing the blood–brain barrier [7].

Leveraging immunology as a therapeutic option for managing central nervous system (CNS) tumors is gaining recognition and momentum [8]. Notable progress has been achieved by using antibody therapy with immune checkpoint inhibitors (ICIs), cellular therapy with adoptive chimeric antigen receptor (CAR) T-cell, vaccine therapy, and oncolytic viruses [9,10]. Remarkable clinical progress has been noted in the deployment of monoclonal antibodies aimed at targeting checkpoint inhibitors to manage advanced brain metastases resulting from melanoma and breast cancer. This observation is particularly intriguing given the resistance of such tumors to conventional chemotherapy [1,2]. CAR-T cells against CCR2b antigen expressed in B cells have potent antitumor activity against non-small cell lung cancer, with notable promise in targeting brain metastases via engineering of the CCL2/CCR2 axis [11]. These examples showcase the potential of systemic immunotherapies to combat CNS malignancies.

Concurrently, in-depth investigation into the microenvironment of brain metastases is continuously advancing the development of mechanism-based immunotherapies to improve clinical outcomes. A novel concept proposes that the occurrence of brain metastases differs significantly from extracranial diseases [12]. This is because the development of cancer cells within the CNS microenvironment in the brain is more intricately illustrated than in other organs. This may be primarily due to the unwelcoming environment for incoming cancer cells in the organ where the cancer initially originated [13,14]. The poor clinical outcomes associated with brain metastases can be attributed to the intrinsic heterogeneity between and within metastatic lesions, along with molecular variations that arise through clonal selection from the primary tumor site [15]. Therefore, understanding the fundamental differences in the biological microenvironment between brain metastases and their originating tumors across various cancer types is crucial for developing effective therapeutic strategies. Furthermore, integrating these observations with evolving surgical and radiotherapy paradigms is a key advancement necessary to improve patient prognosis following a diagnosis of brain metastases.

Current literature has extensively explored the use of immunotherapy for treating brain metastases from various perspectives [16,17]. However, there is still a lack of comprehensive visual analysis and summary with regard to the research trends, primary contributors, and emerging hotspots. Our research herein presents an all-encompassing overview of recent research on immunotherapy for brain metastases, investigating the latest epidemiological, genetic, microenvironmental, leptomeningeal, neurocognitive, targeted therapy, immunotherapy, and prophylactic findings from a range of preclinical and clinical studies. Via bibliometric analysis, we presented a visualized distribution of annual publication, recent developing trends in research keywords, as well as connecting networks among key authors, countries, institutions, and journals. Moreover, our study presents an in-depth analysis of the research areas that are currently thriving and those that are expected to shape the future of the field, based on the keyword outbreaks identified during the research. Finally, we propose plausible solutions to tackle the significant challenges in utilizing immunotherapy to treat brain metastases.

The findings from our study serve as a useful tool for both seasoned and novice professionals in this field of research. It assists in assessing the extent of current research, identifying novel and compelling topics of interest, and formulating strategies for future research. Furthermore, it serves as an essential reference tool for researchers striving to achieve a comprehensive and in-depth understanding of research on immunotherapy for brain metastases.

## 2. Results

### 2.1. Literature Selection Strategy and Conceptual Design of the Entire Study

**Figure 1:**
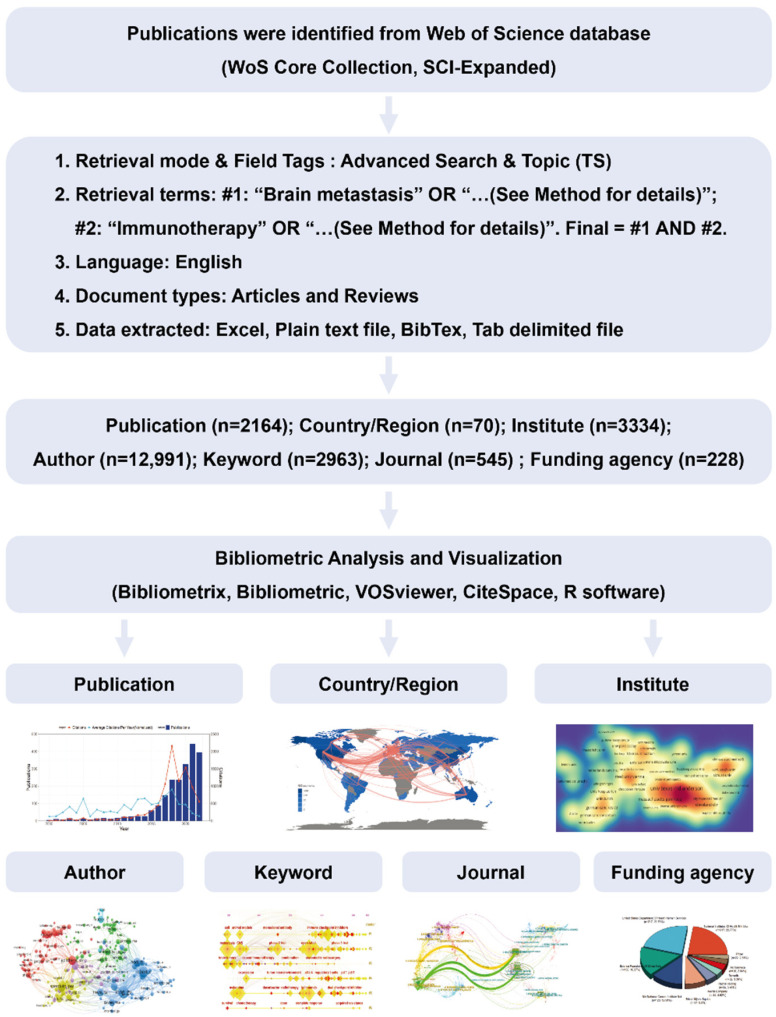
Literature selection strategy and conceptual design of the study.

According to our retrieval strategy, publications in research on immunotherapy for brain metastases were obtained from the Web of Science (WoS) Core Collection electronic database. Retrieved articles were assessed by two researchers independently to avoid bias and remove duplications. The search and selection strategy resulted in a total of 2164 publications, including 1368 original articles and 796 reviews. After preliminary analysis, these publications involved 70 countries, 3334 institutions, 12,991 authors, 2963 keywords, 545 journals, and 228 funding agencies (Figure 1).

### 2.2. Distribution and Cooperation of the Contributing Countries/Regions

**Figure 2:**
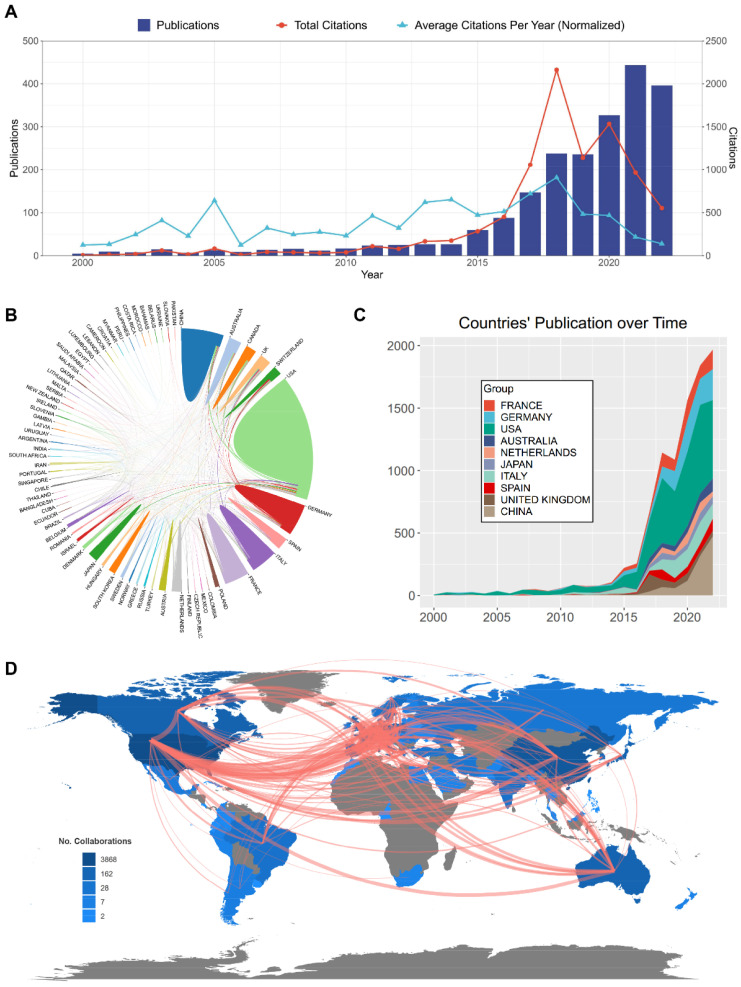
Distribution of countries/regions and the co-operation relations. (**A**) Analysis of annual publications and citation trends from 2000 to 2022. (**B**) The network map visualizing international collaborations across countries. (**C**) The changing trend of the annual publication number in the top 10 countries from 2000 to 2022. (**D**) The world map that visualizing the distribution of countries/regions worldwide and their collaborations, presented in a network format. Red lines indicate the strength of collaboration. This map was downloaded from “Bibliometrix” public online website.

First, we analyzed the trend of publications and calculated total/average citations in the research of immunotherapy for brain metastasis over time (Figure 2A). A regression model was used to depict the time curve of cumulative publication. The number of papers published in this field started to surge in 2014, but it was also from this year onwards that the average citations per year began to decrease annually (Supplementary Figure S1). In the past 22 years, a total of 70 Countries/Regions and 3334 institutions have published papers in regard to immunotherapy for brain metastasis. The top 10 countries with the most publications include the United States (USA), China, Italy, Germany, France, Japan, Australia, Canada, the United Kingdom (UK), and Spain, among which demonstrate extensive collaboration (Figure 2B,C). Notably, the USA and China exhibit the closest collaboration and dominate the field, collectively contributing to over half of global publications (Table 1). Additionally, the links between countries/regions are primarily concentrated between North America and Europe, with strong connections between Oceania and Europe (Figure 2D). A cluster visualization map depicted the distribution of countries/regions and the co-operation relations (Supplementary Figure S2A,B).

### 2.3. Contributing Institutions and Funding Agencies

**Figure 3:**
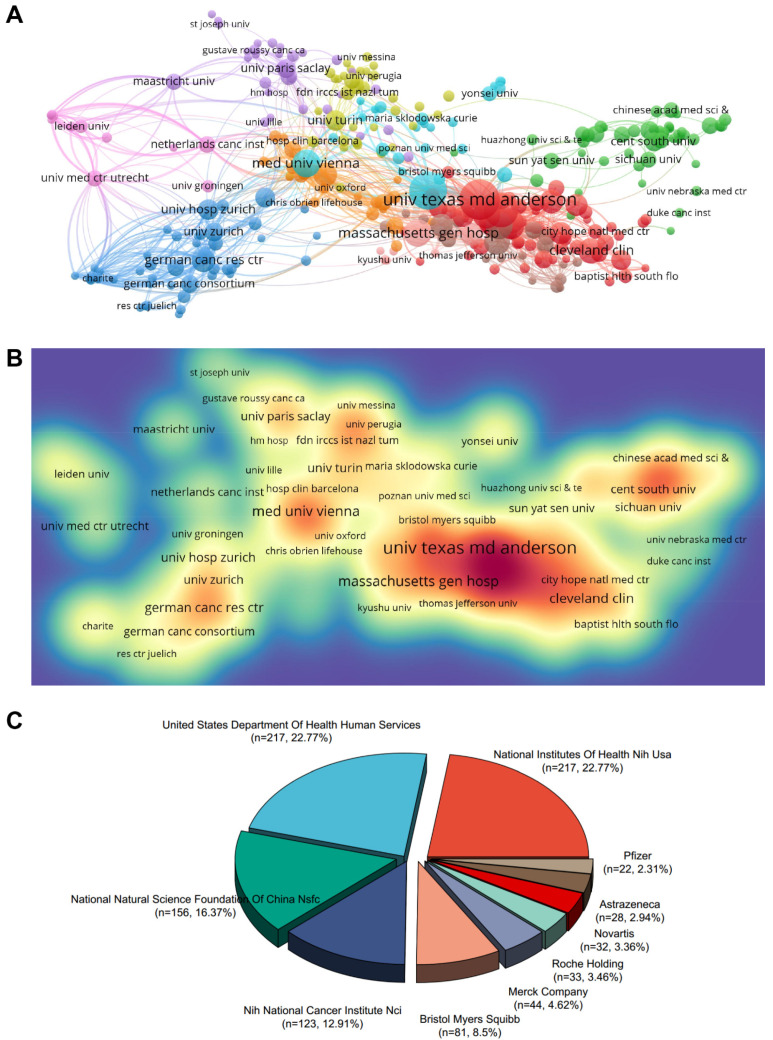
Contribution of productive institutions and funding agencies. (**A**) The VOSviewer visualization map shows institution co-authorship analyses overlaid. The nodes of different colors represent the institutions with different clusters, and the size of the nodes indicates their node sizes. (**B**) Institutions were mapped according to their spectral density. The deeper colors of the nodes represent the higher the number of documents published by the institution. (**C**) The top 10 funding agencies sponsored the highest number of studies in the field of immunotherapy for brain metastases.

Next, we conducted a systematic analysis of productive institutions and funding agencies. According to the results, eight of the top 10 productive institutions in terms of publication volume are from the United States, followed by Germany and Austria (Table 2). ranked first in publications with 90 articles, while Memorial Sloan Kettering Cancer Center ranked first in citations with 3457 times. In addition, the top 3 productive institutions with the highest TLS (Total Link Strength) are M.D. Anderson Cancer Center from The University of Texas (TLS = 62,170), Harvard Medical School (TLS = 48,859), and Memorial Sloan Kettering Cancer Center (TLS = 32,713). From the process via VOSviewer, institutional cooperation forms are divided into eleven closely related clusters (Figure 3A). A density visualization map of institutional cooperation was also displayed (Figure 3B). The 10 most influential funding agencies that support the investigation of immunotherapy for brain metastases are the National Institutes of Health NIH USA, the United States Department of Health Human Services, the National Natural Science Foundation of China NSFC, NIH National Cancer Institute NCI, Bristol Myers Squibb, Merck Company, Roche Holding, Novartis, AstraZeneca, and Pfizer (Figure 3C).

### 2.4. Active Authors and Co-Citation Analysis

**Figure 4:**
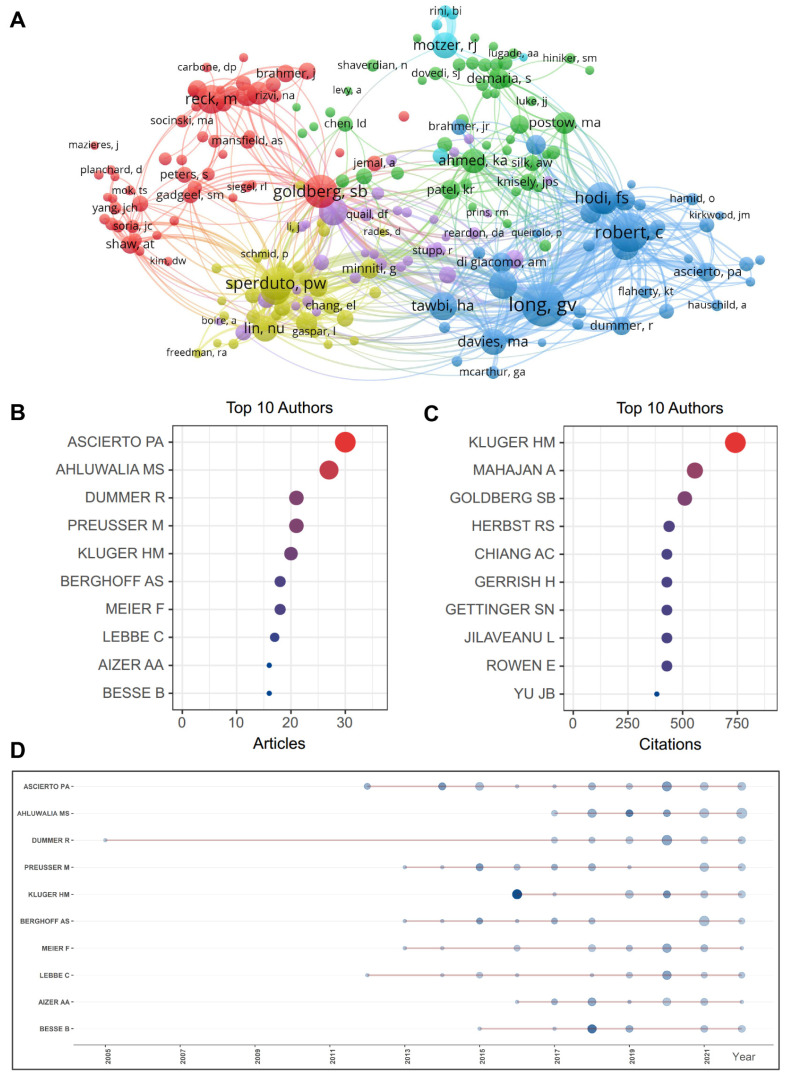
Contribution of active authors. (**A**) Visualization map of authors investigating immunotherapies for brain metastases. The nodes denote authors, with bigger circles representing more publications. Lines between the nodes denote the relationship between authors on the same article, with wider lines representing more frequent collaborations. (**B**) Bubble diagram displaying the most published authors in the field of immunotherapy for brain metastases (related to Table 3 summarizing total citations and h-index of these authors). (**C**) Bubble diagram displaying the most cited authors in the field of immunotherapy for brain metastases. (**D**) Top 5 authors’ production over time is displayed.

Totally, 12,991 authors contributed to the research in the field of immunotherapy for brain metastasis. A visualized cluster map depicted the analysis of author and co-citation (Figure 4A). The scholar who has published the most articles is Ascierto PA (Istituto Nazionale Tumori IRCCS), and the highest cited scholar is Kluger HM (Yale School of Medicine) in the field of immunotherapy for brain metastasis (Figure 4B,C). Furthermore, the five authors with the most publications are displayed in Figure 4D. The top 10 most co-cited authors are Long GV (University of Sydney), Robert C (Gustave Roussy and Paris-Saclay University), Goldberg SB (Yale School of Medicine), Sperduto PW (Duke University Medical Center), Hodi FS (Dana-Farber Cancer Institute), Brown PD (Mayo Clinic), Reck M (German Center for Lung Research), Berghoff AS (Medical University of Vienna), Tawbi HA (University of Texas MD Anderson Cancer Center), and Margolin K (Providence St. John’s Cancer Institute) (Table 3).

### 2.5. Keywords Analysis Regarding Co-Occurrence, Burstiness, Vicissitude, and Clustering

Keywords can be used to analyze the frontiers of immunotherapy for brain metastasis research by providing an overview of the article’s core content. We identified 2963 keywords in total from these publications. Among them, the top 20 keywords with most co-occurrence are displayed in Table 4.

**Figure 5:**
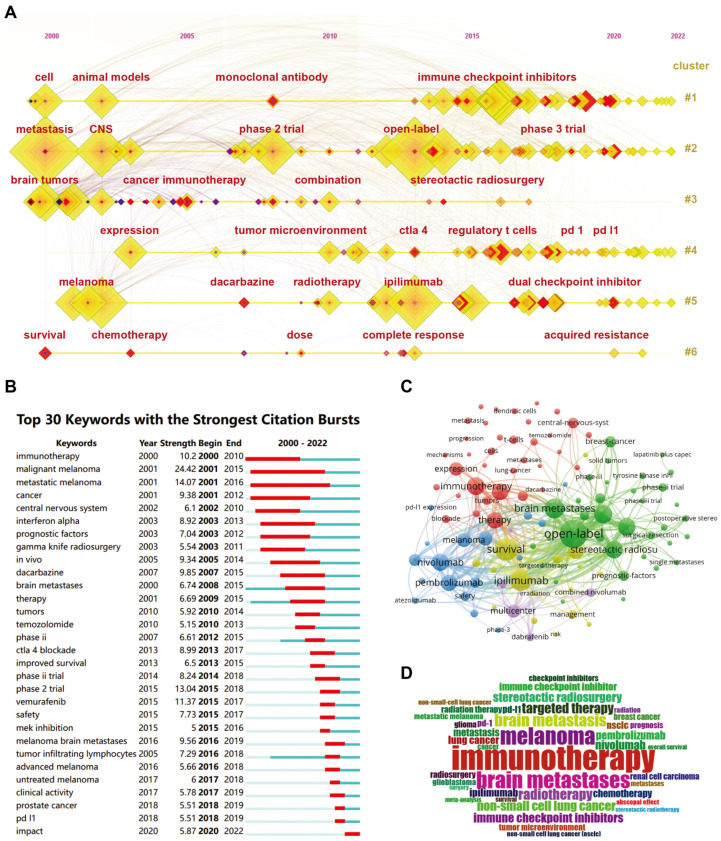
Analysis of keywords co-occurrence and burstiness. (**A**) Visualization map of timeline view of keywords analysis by CiteSpace. (**B**) Timeline distribution of cluster analysis of the top 30 keywords. (**C**) Keywords PLUS analysis with network visualization map of via VOSviewer. (**D**) Keywords representation with word cloud.

**Figure 6:**
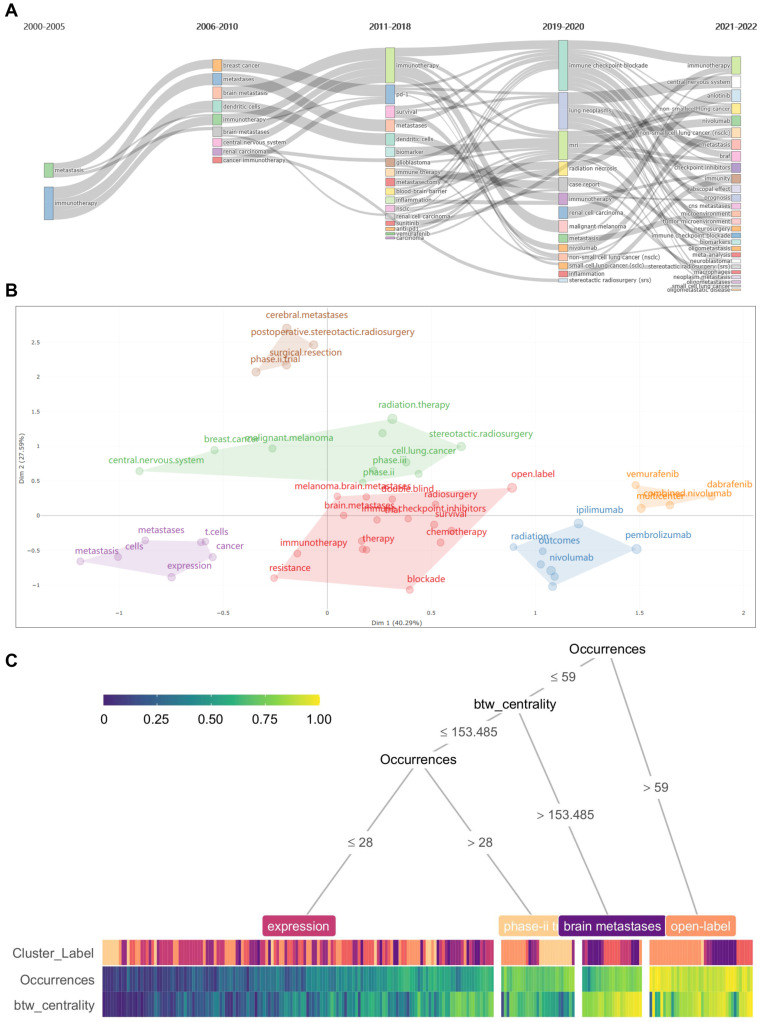
Analysis of keywords vicissitude and clustering. (**A**) The Sankey diagram illustrated the occurrence frequency of keywords over time. (**B**) The keywords in each time period can be divided into six categories. (**C**) Decision tree algorithm revealed that the keywords could be distinguished according to the occurrences and centrality.

According to the results, “Immunotherapy”, “Brain Metastases”, and “Melanoma” are the top 3 keywords, which occur more than 300 times. In addition, we further clustered all the co-occurrence keywords through the timeline view of CiteSpace. All the keywords can be divided into six subclusters with excellent homogeneity (Figure 5A). The citation burst of keywords, which is a method used to identify frequently mentioned keywords during a specific period, is analyzed using CiteSpace (Figure 5B). Among keywords of the strongest citation bursts ranking in the top 30, “malignant melanoma (strength 24.42, 2001–2015), “metastatic melanoma’ (strength 14.07, 2001–2016), “phase 2 trial” (strength 13.04, 2015–2018) are the top three showing the strongest burstiness. Furthermore, we perform a visualized overlay map of keywords together with the analysis of co-occurrence (Figure 5C). Based on the average year of occurrence, keywords are colored accordingly. “Open-label”, “Survival”, and “ipilimumab” are the top three co-occurrence keywords plus. We show the occurrence frequency of these keywords through word cloud analysis (Figure 5D). Next, we analyze the occurrence frequency of keywords over time. Articles published between 2000 and 2005 focused on the risk of metastasis and prognostic factors. Articles published between 2006 and 2010 focused on treating specific types of tumors with brain metastases. The papers published between 2011 and 2018 focus on novel approaches to immunotherapy for tumor brain metastases. Recent publications have focused on clinical trials of the efficacy of various immune checkpoint inhibitors against brain metastases of various tumors (Figure 6A). We re-cluster the keywords in each stage and find that they can be divided into six categories (Figure 6B). We analyzed the theme through the decision tree algorithm and found that these keywords could be distinguished according to their occurrences and centrality (Figure 6C).

### 2.6. Impactful Journals and Co-Citation Analysis

**Figure 7:**
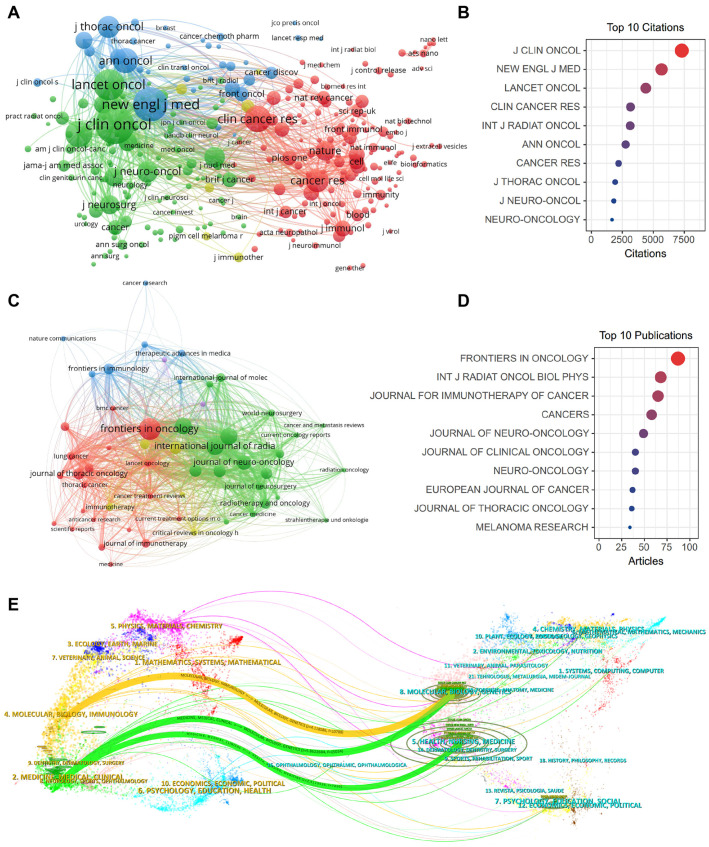
Analysis of influential journals and co-cited journals. (**A**) The network visualization maps of the most influential journals produced with VOSviewer. (**B**) Bubble diagram displaying the most influential journals in the field of immunotherapy for brain metastases. (**C**) The network visualization maps of the most co-cited journals produced with VOSviewer. (**D**) Bubble diagram displaying the most co-cited journals in the field of immunotherapy for brain metastases. (**E**) A biplot overlay of journals with research on immunotherapy for brain metastases. (Left side depicts research fields covered by citing journals, right side shows research fields covered by cited journals).

We next performed a systematic analysis of influential journals and co-cited journals. There are 545 journals regarding immunotherapy for brain metastasis. The ranking of journals with the most published articles is displayed (Table 5). Journals with the most productions and co-citations are *Front Oncol* (IF = 4.7, Publication number = 97) and *J Clin Oncol* (IF = 45.3, Total Citations = 7780), respectively. *New Engl J Med*, with the highest IF of 158.5, ranked first as the most co-cited journal, while *Front Oncol* ranks showed the highest H-index of 56. Furthermore, the top 10 most influential journals or co-cited journals are classified as Q1/2 according to the Journal Citation Reports (JCR) in 2022. The co-citation analysis of journals was depicted via a cluster visualization map. We found that *J Clin Oncol*, *N Engl J Med*, and *Lancet Oncol* are at the core of the co-citation network (Figure 7A,B). The network visualization for the most productive journals revealed that *Front Oncol*, *Int J Radiat Oncol Biol Phys*, and *J Immunother Cancer* are at the central position of the publication network (Figure 7C,D). A dual map overlay revealed the correlation of research disciplines and the citation relationships among the influential journals related to immunotherapy for brain metastases (Figure 7E). 

### 2.7. Influential References and Co-Citation Analysis

**Figure 8:**
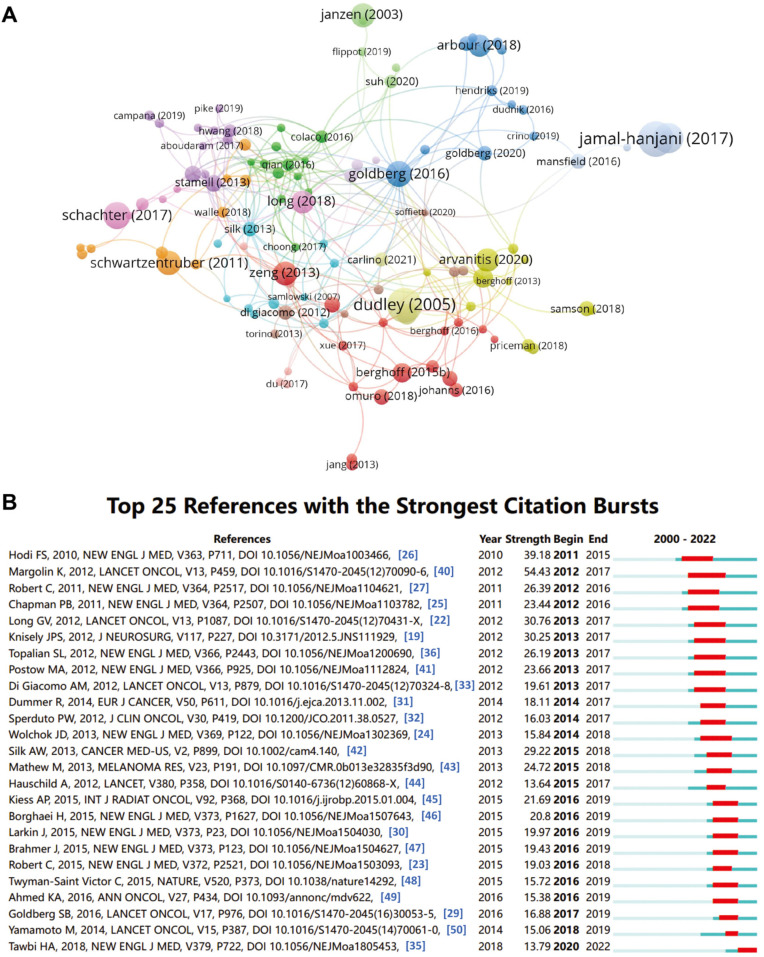
Analysis of references and co-cited references. (**A**) The network visualization for the most co-cited references in the field of immunotherapy for brain metastases produced with VOSviewer. The red line represents the time interval of the citation burst. (**B**) Top 25 references with the strongest citation bursts in the field of immunotherapy for brain metastases. (Hodi FS, 2010 [26]; Margolin K, 2012 [40]; Robert C, 2011 [27]; Chapman PB, 2011 [25]; Long GV, 2012 [22]; Knisely JPS, 2012 [19]; Topalian SL, 2012 [36]; Postow MA, 2012 [41]; Di Giacomo AM, 2012 [33]; Dummer R, 2014 [31]; Sperduto PW, 2012 [32]; Wolchok JD, 2013 [24]; Silk AW, 2013 [42]; Mathew M, 2013 [43]; Hauschild A, 2012 [44]; Kiess AP, 2015 [45]; Borghaei H, 2015 [46]; Larkin J, 2015 [30]; Brahmer J, 2015 [47]; Robert C, 2015 [23]; Twyman-Saint Victor C, 2015 [48]; Ahmed KA, 2016 [49]; Goldberg SB, 2016 [29]; Yamamoto M, 2014 [50]; Tawbi HA, 2018 [35]).

Finally, we analyzed the most influential references in the field of immunotherapy for brain metastasis. Articles with the highest citations ranked in the top 10 are summarized in Table 6. The most influential literature in the field of immunotherapy for brain metastasis was contributed by Sarah B Goldberg et al. in 2016 to *Lancet Oncol*, with a total citation count of 342 times. The network visualization for the most co-cited references revealed that dudley (2005), goldberg (2016), and zeng (2013) are at the core of the publication co-citation network (Figure 8A). Hot-cited literature has been explored in recent years using references with citation bursts, an evaluation method that can reflect the relationship between citation volume. Therefore, we also analyzed the top 25 references with the strongest citation bursts (Figure 8B). For articles published within 2010–2012, there has been an explosion of citations to articles on immunotherapy for brain metastasis, which started in 2011. Generally, over the past ten years, most articles are still cited frequently, indicating that immunotherapy for brain metastasis research continues to flourish. 

**Table 6:** Top 10 co-cited research articles regarding immunotherapy for brain metastases.

Rank	Title	First Author	Institute (Country)	Year	Journal	IF (2023)	TLS	Citations (Ref.)
1	Pembrolizumab for patients with melanoma or non-small-cell lung cancer and untreated brain metastases: early analysis of a non-randomized, open-label, phase 2 trial	Sarah B Goldberg	Yale School of Medicine (USA)	2016	*Lancet Oncol*	51.1	4142	342 [29]
2	Ipilimumab in patients with melanoma and brain metastases: an open-label, phase 2 trial	Kim Margolin	Providence Saint John’s Health Center (USA)	2012	*Lancet Oncol*	51.1	4061	327 [40]
3	Combined Nivolumab and Ipilimumab in Melanoma Metastatic to the Brain	Hussein A Tawbi	University of Texas, MD Anderson Cancer Center (USA)	2018	*N Engl J Med*	158.5	3309	291 [35]
4	Improved survival with ipilimumab in patients with metastatic melanoma	F Stephen Hodi	Dana–Farber Cancer Institute (USA)	2010	*N Engl J Med*	158.5	2972	264 [26]
5	Combination nivolumab and ipilimumab or nivolumab alone in melanoma brain metastases: a multicentre randomized phase 2 study	Georgina V Long	University of Sydney (Australia)	2018	*Lancet Oncol*	51.1	3005	246 [20]
6	Nivolumab versus Docetaxel in Advanced Nonsquamous Non-Small-Cell Lung Cancer	Hossein Borghaei	Fox Chase Cancer Center (USA)	2015	*N Engl J Med*	158.5	2260	226 [46]
7	Pembrolizumab versus Chemotherapy for PD-L1-Positive Non-Small-Cell Lung Cancer	Martin Reck	German Center of Lung Research (Germany)	2016	*N Engl J Med*	158.5	2111	196 [38]
8	Stereotactic radiosurgery for melanoma brain metastases in patients receiving ipilimumab: safety profile and efficacy of combined treatment	Kiess A.P	Memorial Sloan-Kettering Cancer Center (USA)	2015	*Int J* *Radiat Oncol Biol Phys*	7.0	2633	178 [45]
9	Combined Nivolumab and Ipilimumab or Monotherapy in Untreated Melanoma	Larkin J.	Memorial Sloan-Kettering Cancer Center (USA)	2015	*N Engl J Med*	158.5	1839	161 [30]
10	Nivolumab versus Docetaxel in Advanced Squamous-Cell Non-Small-Cell Lung Cancer	Brahmer Julie	Sidney Kimmel Comprehensive Cancer Center at Johns Hopkins (USA)	2015	*N Engl J Med*	158.5	1628	157 [47]

IF, Impact Factor; TLS, Total Link Strength.

## 3. Discussion

### 3.1. General Information

Distinct from standard review articles or meta-analyses, bibliometric analysis enlists distinctive merits in comprehensively encapsulating the progression trajectory of particular research domains as well as pinpointing pertinent research avenues. The present research constitutes a pioneering effort in conducting a knowledge structure analysis and identifying the plausibility of forthcoming research frontiers concerning immunotherapy for brain metastasis via the bibliometric methodology. Additionally, completed and ongoing clinical trials to evaluate the effectiveness of immunotherapy for brain metastases are summarized in Table 7 and Table 8, respectively.

North America, Europe, and Asia, as outlined in Figure 2, dominate research in immunotherapies for brain metastases, with the United States emerging as the leading contributor, with 80% of the top 10 institutes publishing related studies, a higher number of publications, TLS, and H-index than other countries/regions. Encouragingly, China, the second most productive country in this domain, has registered a remarkable rise in its research output since 2019, suggesting that developing countries’ interest has contributed positively to the rapid advancement of immunological research in brain metastases. Collaborative initiatives between leading players such as the US, at the forefront of cutting-edge research, and developing countries like China, with significant clinical and experimental cases, could optimize the potential efficacy of immunotherapy in managing brain metastases. Moreover, collaborative networks play a crucial role in enhancing research quality by facilitating the exchange of knowledge, resources, and expertise. Through such collaboration, researchers can pool their intelligence and practical insights to address complex research inquiries that may surpass the capabilities of a single institution. Consequently, this fosters the production of more impactful research with the potential to shape policies and practices. Additionally, cooperation among elite institutions contributes to the broader dissemination and visibility of research outcomes. These institutions often possess well-established networks and partnerships with other organizations, enabling wider dissemination of research findings. As a result, this heightened visibility leads to increased recognition and impact of the research, while also creating more opportunities for future collaborations and funding.

Of note, Dr. Manmeet Singh Ahluwalia of the Miami Cancer Institute led the authorship ranks with 24 publications and 881 citations, followed by Dr. Harriet Kluger of Yale School of Medicine and Dr. Matthias Preusser at the Medical University of Vienna, notable for their contributions on melanoma. In light of *Frontiers in Oncology* being the journal with the most pertinent articles and *Lancet Oncology* containing the most cited paper, these journals are being suggested for future reference in practice and research.

### 3.2. Keywords and Emerging Hotpots

In light of clusters and timeline views illuminating the cardinal themes and key topics of immunotherapy for brain metastases, the identified leading hotspots can be succinctly distilled into the following themes. These include elucidating the mechanisms of immune evasion in brain metastases, optimizing treatment strategies for patients with brain metastases, and identifying reliable biomarkers that can predict response to immunotherapy in these patients. Furthermore, researchers should aim to investigate the potential of combination therapies that can synergistically enhance the efficacy of immunotherapy in treating brain metastases.

Immunotherapy, as the primary keyword, has fueled substantial recent growth in publications related to brain metastases, with a significant emphasis on immune checkpoint inhibitors (ICIs) [16]. This intersection between the immune system and brain metastases is a fascinating and expanding area with potential clinical significance, particularly when regarding the lymphatic structure [57,58]. The majority of ongoing clinical trials have strongly favored ICIs as a viable therapeutic strategy for brain metastases. Evidence suggests that immunomodulatory factors like PD-1, PD-L2, and other cytokines are regularly expressed in brain metastases originating from breast and lung carcinoma, as well as melanoma [28,59,60]. A noticeable discrepancy between paired primary tumors and brain metastases is significant in the inflammatory microenvironment of patients with melanoma [61,62]. Analysis of lung cancer patients reveals that tumor cell PD-L1 expression differed in 14% of cases, while TIL PD-L1 expression exhibited differences in over one-fourth of cases. Interestingly, some brain metastases lack TIL infiltration, PD-L1 expression, or both, which are found in the primary sites of lung cancer, despite their origins [63]. 

Encouragingly, a phase II trial investigating the CTLA-4 inhibitor ipilimumab for patients with brain metastases has produced satisfactory findings [40]. Additionally, over one-fourth of the patients experienced OS after two years, indicating the prolonged benefit of immunotherapy to a specific subset of patients. However, patients who showed symptoms and were on steroid treatment at the beginning of follow-up had bleak outcomes generally, but still, one in ten of those patients survived for more than two years. There are ongoing studies of PD-1 inhibitors for brain metastases, which have already demonstrated encouraging and long-lasting activity in several cancers, including those that originated from the skin, bladder, and lung. In a recent phase 2 trial that involved participants with progressing but yet asymptomatic brain metastases from non-small-cell lung cancer (NSCLC) or melanoma, pembrolizumab (a PD-1 inhibitor) exhibited prominent and enduring activity in CNS for both malignancies [29]. Among the 18 participants diagnosed with melanoma, four of them showcased an intracranial response. On the other hand, four out of the 18 NSCLC participants exhibited a complete response in the brain malignant regions. During the data analysis, a significant proportion of patients exhibited long-lasting and persistent responses to the treatment. Moreover, the impact of nivolumab, a PD-1 inhibitor, on NSCLC patients with untreated CNS metastases was also evaluated. Encouragingly, positive intracranial responses were observed in two out of the twelve participants who received nivolumab [64]. Furthermore, nivolumab, another ICI of PD-1, has been administered to participants with lung cancer and untreated CNS metastases. The preliminary report indicates that one-sixth of the patients displayed a complete response within the CNS area, one of whom also achieved a complete response after nearly one year [64].

From the perspective of clinical practice, though immunotherapy has shown positive, albeit restricted, outcomes in patients with brain metastases, fundamental queries persist. Additional mechanistic investigations into the therapeutic action of the brain area are required to address the question of whether they function locally or react systemically to immune stimulation. Augmenting preclinical observations in mechanic research to further comprehend the potential anti-tumor impacts of immunotherapies is crucial to enhancing the benefits of this therapy and developing more efficient treatments.

From the perspective of molecular biology, recent studies have investigated the microenvironmental characteristics of primary and metastatic brain tumors via transcriptomic and proteomic approaches. Although stromal cell composition was uniform across studies, discrepancies in the constitution and expression of immune cells were observed amongst various brain tumors [65]. Enrichment in distinct immune cell types was observed in different metastatic tumors, suggesting that CNS metastases shape their microenvironment differently from their extracranial origins [66]. These investigations prime the knowledge of the microenvironment’s unique cell composition for different diseases at the same anatomic site and highlight the inadequacy of the current generalized therapies used to mediate the tumor microenvironment. Furthermore, these studies demonstrate that the microenvironment is not static or uniform [67]; even though the homeostatic niche of organs may be similar at the beginning, cancer cells infiltrate and cause the local evolution in a synergic pattern, leading to the recruitment of immunologic components with regard to the specific type of disease and related cells. 

It is probable that further insights into the principles of neurology-immunology-oncology crosstalk will be the trend to come. One potential avenue of exploration is the potential role of neuroscience drugs in ameliorating the immune-suppressive tumor microenvironment and enhancing immuno-oncology strategies. For instance, it is conceivable that by targeting the signaling pathway of neurological modulators or transmitters, the immunologic microenvironment could be altered to facilitate an anti-tumor immunologic response [68]. A comprehensive advancement in the mechanisms behind the interactions between the CNS, the immune system, microbes, and cancer could offer valuable new perspectives in developing immuno-oncology strategies [69].

Beyond the investigation of immunotherapy for brain metastases via clinical trial and molecular research alone, these ongoing studies propose a possible pathway to align them for better therapeutic practice: to implement targeted immunotherapies customized to the genomics of brain metastasis. It is possible that immunotherapies for metastases may differ from primary tumor reactions due to significant molecular pathway alterations. However, by conducting genomic profiling of the metastatic compartment, new therapeutic strategies can be devised, clinical response predictions can be made, and new intervention targets can be identified. Currently, there has been a surge in the utilization of single-cell transcriptomics and computational systemic biological techniques, which have enabled the comprehensive characterization of microenvironmental changes and clonal dynamics in unprecedented detail and scale. New and emerging techniques have opened up fresh avenues for analyzing epigenetic markers, proteins, and metabolites at a single-cell and spatial level [70,71]. Recent developments in DNA-editing technology have led to the creation of inducible lineage recording functions with high fidelity, which enable accurate state transition of cells over time [72,73]. Together with existing methodologies, such as mitochondrial analysis related to mutation and real-time clonal tracking based on liquid biopsy; these systems are increasingly being used in clinical settings to elucidate the sequence of tumor evolution [74]. Clinical trials utilizing ex vivo models are being considered as potential pointers prior to treatment, aiming at the prediction of patient-specific responses to treatment and providing guidance for clinical decision-making, as evidenced by current prospective studies [75]. Last but not least, the use of artificial intelligence is expected to revolutionize the field of clinical trial design, expediting biomarker identification and drug development [76].

### 3.3. Limitations and Future Direction

Immunotherapy has been a game-changer in cancer treatment; however, its impact on patients with brain metastases is yet to be comparable. While present knowledge can aid both research and clinical practice in improving cancer patients’ chances, the unique immunological and clinical features of brain metastases present significant challenges. These features include distinctive genetic and epigenetic alterations from the primary tumors and unique immune microenvironments likely to have an impact on the response to immunotherapies [77,78]. It is thus critical to focus on developing improved preclinical models, rational assays, and intensive early phase clinical trials to advance immunotherapy for brain metastases and understand neurological toxicity.

With the rapid evolution of personalized therapies, innovation must be embraced with flexible designs that incorporate biomarkers and robust decision-making [79]. A collective effort from all stakeholders, which includes philanthropic organizations, governmental bodies, and other funding bodies, is crucial to addressing the existing funding gaps. The primary objective of this collaborative research is to boost patient-oriented basic and clinical investigation, educate patients and society, as well as facilitate interdisciplinary collaboration among scientists and physicians to improve patient outcomes. Given the complex outcomes observed in immunotherapeutic trials for cancer patients with brain metastases, close academic collaboration among all disciplines is gaining importance. Progress at the interface of these key participants’ interactions is necessary for significant advancements. 

In spite of the comprehensive landscape presented in this study on immunotherapy for brain metastases, the analysis was exclusively conducted with the WoS Core Collection electronic database because of its emphasis on high-quality, peer-reviewed research while excluding extraneous and quasi-experimental studies. However, it is worth considering the potential merits of exploring additional databases that encompass a broader range of biomedical research, including conference proceedings and non-peer-reviewed papers. Such an exploration could offer supplementary insights with clinical implications, warranting further investigation in these domains.

## 4. Materials and Methods

### 4.1. Database and Study Collection

The WoS Core Collection electronic database (Clarivate Analytics, Philadelphia, PA, USA) was used to retrieve related literature published between 1965 and 2023, according to the following search strategy: #1: Topic = (“Brain metastasis” OR “Brain metastases” OR “Central nervous system metastases” OR “Intracranial metastasis” OR “Cerebral metastasis”); #2: Topic = (“Immunotherapy” OR “Immune checkpoint therapy” OR “Immune checkpoint inhibitor”); #Final data source: #1 AND #2. Only publication in forms of article and review, present in English, were included for further analysis. Research bias was avoided by conducting the literature search independently by two researchers scrutinizing relevant articles and reviews on 20 April 2023. There was a restriction on language to English only. A flowchart for this study is presented in Figure 1.

### 4.2. Visualization and Statistical Analysis

Bibliometric visualization is commonly conducted with VOSviewer software to create maps that portray knowledge structures and networks [80]. The three most prominent visualization maps offered by VOSviewer include maps of network visualization. VOSviewer (Version 1.6.16) was applied in this study to perform an analysis of the co-authorship (regarding authors, countries, and institutions) and the co-citation of journals. Keywords occurring more than 20 times were utilized in co-occurrence network analysis to identify the prevailing terms in research on immunotherapy for brain metastases.

CiteSpace (Version 6.2.R2), a prominent visualization tool created by Professor Chaomei Chen [81], was employed to generate analysis maps for the co-citation of references and authors, as well as to identify the keywords that exhibit the most substantial citation spikes in research on immunotherapy for brain metastases. An overlay dual-map of journals was also generated via CiteSpace. Parameters used in CiteSpace were as follows: Year of slice, 1; Selection criteria, Top 50; Link retaining factor, 3; Look back years, 8; e for top N, 2; Pruning, Pathfinder. Additionally, both the online bibliometric platform (website: http://bibliometric.com/) and the “Bibliometrix” package for R-software (Version 4.2.3) were utilized to execute an analysis on international collaboration. The graphical representation of the data was predominantly performed with the VOSviewer and CiteSpace visualization tools.

## 5. Conclusions

In summary, the treatment options available for brain metastases have been substantially broadened by blending clinical insights with innovative biological research. The adoption of multimodal, interdisciplinary approaches that enhance treatment outcomes will tremendously benefit patients suffering from cancer-related brain metastases. With continued collaboration and advancement in this field, we can seek to make further improvements in the management of this challenge, ultimately leading to better health outcomes for affected individuals.

## Figures and Tables

**Table 1:** Top 10 productive countries in research of immunotherapy for brain metastasis.

Rank	Country	Counts	Total (%)	CAF (%)	ACI	Total Citations
1	USA	1017	47.0	15.7	32.32	32,868
2	China	340	15.7	16.0	11.20	3808
3	Germany	217	10.0	22.0	35.57	7719
4	Italy	209	9.7	19.3	26.19	5474
5	France	158	7.3	24.7	25.07	3962
6	Canada	110	5.1	23.4	38.60	4246
7	Japan	104	4.8	2.7	17.91	1863
8	UK	102	4.7	22.0	62.05	6329
9	Spain	99	4.6	30.0	37.70	3732
10	Australia	97	4.5	22.0	47.51	4608

CAF, Corresponding Author Frequency; ACI, Average Citations per Item.

**Table 2:** Top 10 institutes with most publication related to immunotherapy for brain metastasis.

Rank	Institutes	Country	Counts	TLS	ACI	Total Citations
1	Univ Texas Md Anderson Canc Ctr	USA	90	62,170	31.31	2818
2	Harvard Med Sch	USA	79	48,859	33.35	2635
3	Mem Sloan Kettering Canc Ctr	USA	67	32,713	51.59	3457
4	Med Univ Vienna	Austria	47	28,921	32.63	1534
5	Dana Farber Canc Inst	USA	45	32,743	58.17	2618
6	Massachusetts Gen Hosp	USA	45	24,676	34.04	1532
7	Mayo Clin	USA	43	26,405	53.04	2281
8	Emory Univ	USA	40	22,998	45.65	1826
9	Univ Pittsburgh	Germany	40	17,970	32.25	1290
10	Cleveland Clin	USA	39	27,931	32.94	1285

TLS, Total Link Strength; ACI, Average Citations per Item.

**Table 3:** Top 10 productive authors and co-authors in research of immunotherapy for brain metastasis.

Rank	Author [Ref.]	Institute (Country)	Counts	Total Citations	H- Index	TLS	Co-Cited Author [Ref.]	Institute (Country)	Total Citations	TLS
1	Ahluwalia, Manmeet S. [3,6,16]	Florida International University (USA)	24	881	44	15,704	Long, GV [18,19,20,21,22,23]	University of Sydney (Australia)	769	12,794
2	Kluger, Harriet M. [19,24]	Yale School of Medicine (USA)	19	1522	59	9712	Robert, C [23,25,26,27]	Gustave Roussy and Paris-Saclay University (France)	638	10,264
3	Preusser, Matthias [16,28]	Medical University of Vienna (Austria)	19	1091	72	9900	Goldberg, SB [16,18,29]	Yale School of Medicine (USA)	498	7585
4	Dummer, Reinhard [30,31]	University Hospital Zurich (Switzerland)	16	132	123	4799	Sperduto, PW [32]	Duke University Medical Center (USA)	483	8658
5	Ascierto, Paolo A. [22,25,33,34]	Istituto Nazionale Tumori IRCCS (Italy)	15	965	96	5369	Hodi, FS [26,35,36]	Dana-Farber Cancer Institute, Harvard Medical School (USA)	453	7332
6	Chiang, Veronica L. [18,19,29]	Yale School of Medicine (USA)	15	618	31	6122	Brown, PD [7,20,21,32]	Mayo Clinic (USA)	405	7305
7	Lauko, Adam [37]	Lerner Research Institute, Cleveland Clinic (USA)	14	58	9	4069	Reck, M [38]	German Center for Lung Research (Germany)	381	4638
8	Aizer, Ayal A. [5]	Dana-Farber Cancer Institute (USA)	13	494	39	8629	Berghoff, AS [28]	Medical University of Vienna (Austria)	376	5521
9	Brastianos, Priscilla K. [7,16,39]	Harvard Medical School (USA)	13	332	49	9979	Tawbi, HA [35]	University of Texas, MD Anderson Cancer Center (USA)	366	5635
10	Heimberger, Amy B.	Northwestern University (USA)	13	255	66	4509	Margolin, K [40]	Providence Saint John’s Health Center (USA)	364	6182

TLS, Total Link Strength.

**Table 4:** Top 20 co-occurrence keywords on research of immunotherapy for brain metastasis.

Rank	Keywords	Occurrences	TLS	Rank	Keywords	Occurrences	TLS
1	Immunotherapy	742	1450	11	Pembrolizumab	87	232
2	Brain Metastases	358	770	12	Lung Cancer	81	182
3	Melanoma	348	706	13	Ipilimumab	78	234
4	Brain Metastasis	200	420	14	Metastasis	77	119
5	Radiotherapy	149	352	15	Chemotherapy	73	172
6	Targeted Therapy	132	315	16	Immune Checkpoint Inhibitor	72	125
7	Non-Small Cell Lung Cancer	122	241	17	NSCLC	72	140
8	Immune Checkpoint Inhibitors	112	223	18	PD-L1	65	160
9	Stereotactic Radiosurgery	112	292	19	PD-1	58	172
10	Nivolumab	98	272	20	Tumor Microenvironment	57	84

TLS, Total Link Strength.

**Table 5:** Top 10 journals with most publication and co-citation in research of immunotherapy for brain metastasis.

Rank	Journals	Counts	IF (2023)	JCR (2023)	H-Index	Total Citations	Co-Cited Journals	IF (2023)	JCR (2023)	Total Citations
1	*Front Oncol*	97	4.7	Q2	56	987	*J Clin Oncol*	45.3	Q1	7780
2	*Int J Radiat Oncol Biol Phys*	68	7.0	Q1	34	1732	*New Engl J Med*	158.5	Q1	5935
3	*J Immunother Cancer*	67	10.9	Q1	50	1175	*Lancet Oncol*	51.1	Q1	4676
4	*Cancers*	65	5.2	Q2	54	611	*Clin Cancer Res*	11.5	Q1	3359
5	*J Neuro-Oncol*	52	3.9	Q2	22	853	*Int J Radiat* *Oncol Biol Phys*	7.0	Q1	3319
6	*J Clin Oncol*	41	45.3	Q1	84	2432	*Ann Oncol*	50.5	Q1	2991
7	*Neuro-Oncology*	41	15.9	Q1	37	1215	*Cancer Res*	11.2	Q1	2365
8	*Eur J Cancer*	40	8.4	Q1	42	865	*J Thorac Oncol*	20.4	Q1	2158
9	*J Thorac Oncol*	37	20.4	Q1	51	907	*J Neuro-Oncol*	3.9	Q2	1960
10	*Melanoma Res*	35	2.2	Q3	13	598	*Neuro-Oncology*	15.9	Q1	1843

IF, Impact Factor; JCR, Journal Citation Reports.

**Table 7:** Key clinical trials assessing immunotherapy for cancer patients with brain metastases.

Tumor Type	Phase	Drugs (Target)	*n*	Trial arm	Intracranial RR (%)	Median PFS	Median OS	Trial No. (Ref.)
Melanoma	I	Ipilimumab (CTLA-4)	17	Ipilimumab + SRS/WBRT	65	2.5 mos	8.0 mos	NCT 01703507 [45]
Melanoma	I	Nivolumab (PD-1)	17	Nivolumab + SRS	60	/	/	NCT 02716948 [51]
Melanoma	II	Ipilimumab (CTLA-4)	72	(1) Asymptomatic: Ipilimumab; (2) Symptomatic: Ipilimumab	(1) 25 (2) 10	(1) 1.9 mos (2) 1.2 mos	(1) 7.0 mos (2) 3.7 mos	NCT 00623766 [40]
Melanoma	II	Nivolumab Ipilimumab (PD-1/CTLA-4)	90	Nivolumab + Ipilimumab induction followed by Nivolumab maintenance	57	59% at 9 mos	82% at 9 mos	NCT 02320058 [35]
Melanoma	II	Ipilimumab (CTLA-4)	86	Ipilimumab + Fotemustine	35	3.0 mos	12.7 mos	NCT 01654692 [33,34]
Melanoma	II	Nivolumab Ipilimumab (PD-1/CTLA-4)	76	(1) Asymptomatic: Nivolumab + Ipilimumab; (2) Asymptomatic: Nivolumab; (3) Symptomatic: Nivolumab	(1) 44 (2) 20 (3) 6	(1) 50% (2) 29% (3) 0% at 6 mos	(1) 75% (2) 59% (3) 44% at 6 mos	NCT 02374242 [21]
Melanoma/NSCLC	II	Pembrolizumab (PD-1)	65	Pembrolizumab	22 33	2.0 mos	17.0 mos	NCT 02085070 [18]
NSCLC/RCC	II	Nivolumab (PD-1)	26	Nivolumab + SRS	42	6.1 mos	21.4 mos	NCT 02978404 [52]
Breast Cancer	I	Nivolumab (PD-1)	14	Nivolumab + SRS	55	/	/	NCT 03807765 [53]
Melanoma	II	Nivolumab Ipilimumab (PD-1/CTLA-4)	128 ^a^	Nivolumab + Ipilimumab followed by Nivolumab or Nivolumab + SRS	/	/	/	NCT03340129 [54]
NSCLC	III	Pembrolizumab (PD-1)	108	Pembrolizumab + Chemotherapy	/	/	/	NCT 02578680 [55]
NSCLC	III	Atezolizumab (PD-L1)	124	Atezolizumab	/	/	16.0 mos	NCT 02008227 [56]
Solid Tumors	II	Pembrolizumab (PD-1)	101	Pembrolizumab ± SRS	40	/	/	NCT 02886585 [39]

^a^, Estimated Enrollment; mos, months; NSCLC, non-small-cell lung cancer; RCC, renal cell carcinoma; RR, response rate; PFS, progression-free survival; OS, overall survival; SRS, stereotactic radiosurgery; WBRT, whole brain radiotherapy.

**Table 8:** Clinical trials underway to evaluate the effectiveness of immunotherapy for brain metastases.

Trial No.	Tumor Type	Phase	Drugs	*n*	Trial Arm	Country	Principle Institute	Duration
NCT 02460068	Melanoma	III	Nivolumab Ipilimumab	168	(1) Ipilimumab + Fotemustine; (2) Ipilimumab + Nivolumab; (3) Bevaczumab + Pembrolizumab; (4) Fortemustine	USA	University Hospital of Siena	2012–2020
NCT 02681549	Melanoma NSCLC	II	Pembrolizumab	53	Pembrolizumab + Bevacizumab	USA	Yale University	2016–2024
NCT 02696993	NSCLC	II	Nivolumab Ipilimumab	88	Nivolumab ± Ipilimumab + SRS or WBRT	USA	M.D. Anderson Cancer Center	2016–2023
NCT 02978404	NSCLC RCC	II	Nivolumab	60	Nivolumab + SRS	Canada	University of Montreal Health Centre	2017–2023
NCT 03340129	Melanoma	II	Nivolumab Ipilimumab	218	Nivolumab + Ipilimumab ± SRS	Australia	Melanoma Institute Australia	2017–2025
NCT 03955198	Melanoma	II	Durvalumab	100	Radiotherapy ± Durvalumab	France	Institut Claudius Regaud	2021–2025
NCT 03175432	Melanoma	II	Atezolizumab bevacizumab	60	Atezolizumab + Bevacizumab ± Cobimetinib	USA	M.D. Anderson Cancer Center	2017–2023
NCT 03873818	Melanoma	I	Ipilimumab Pembrolizumab	30	Ipilimumab + Pembrolizumab	USA	M.D. Anderson Cancer Center	2019–2023
NCT 03696030	Breast Cancer	I	/	39	HER2-CAR T cells	USA	City of Hope Medical Center	2018–2023
NCT 02442297	Breast Cancer	I	/	28	HER2-specific T cells	USA	Baylor College of Medicine	2016–2036
NCT 03449238	Breast Cancer	I/II	Pembrolizumab	41	Pembrolizumab + SRS	USA	Weill Medical College of Cornell University	2018–2026

## Data Availability

All data used and/or analyzed in the current study are available upon reasonable request to the corresponding author.

## Supplementary Materials

The following supporting information can be downloaded at: https://www.mdpi.com/article/10.3390/ph17070850/s1, Figure S1. Trends of annual publications, total citation, and average citations per year in research of cancer immunotherapy for brain metastases. Figure S2. Analysis of collaborative network visualization of countries/regions in VOSviewer. The figure shows the countries/regions with more than 1 number of documents. The nodes of different colors represent the countries/regions with different clusters, and the size of the nodes indicates their node sizes.
