# Supplementary material for: Evolutionary Trend Analysis of Research on Immunotherapy for Brain Metastasis Based on Machine-Learning Scientometrics
Source: Pharmaceuticals (Basel). 2024 Jun 28;17(7):850. doi: 10.3390/ph17070850 (PMC11280367; doi:10.3390/ph17070850)
Supplement: Supplementary file 1 [file pharmaceuticals-17-00850-s001.zip › pharmaceuticals-3046450-supplementary.pdf]

Supplementary Materials

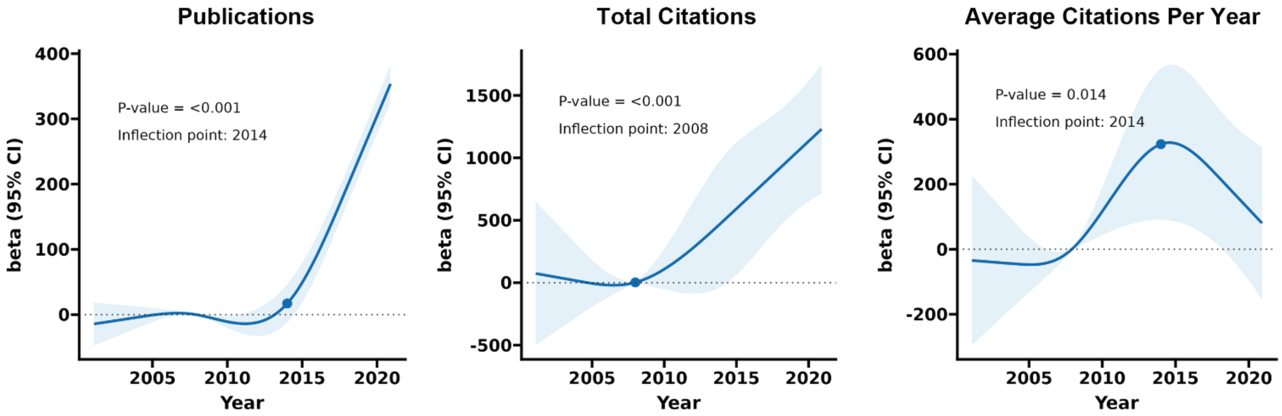

Supplementary Figure S1. Trends of annual publications, total citation, and average citations per year in research of cancer immunotherapy for brain metastases.

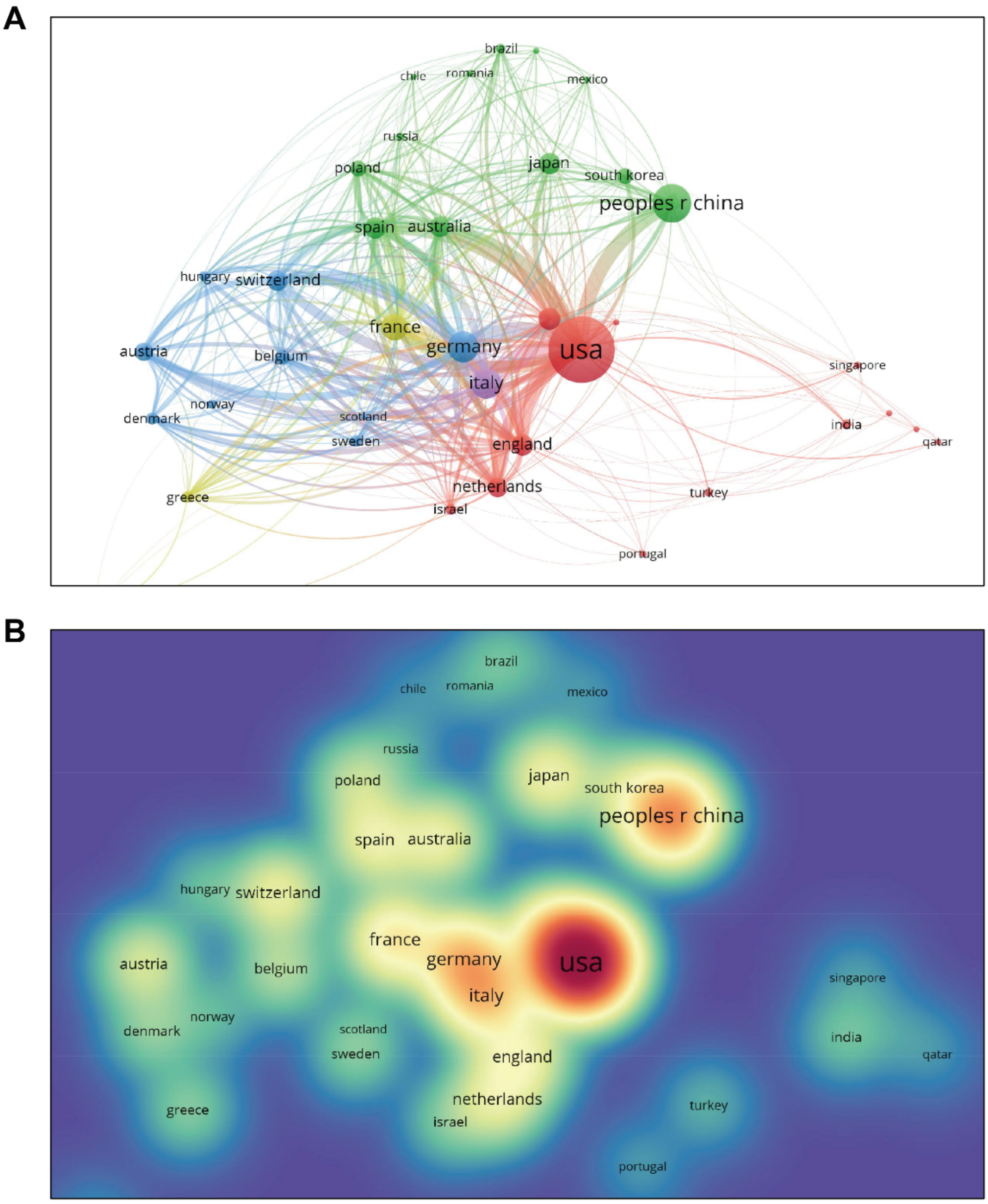

Supplementary Figure S2. Analysis of collaborative network visualization of countries/regions in VOSviewer. (A) The figure shows the countries/regions with more than 1 number of documents. The nodes of different colors represent the countries/regions with different clusters, and the size of the nodes indicates their node sizes. (B) The heatmap shows the number of publications by the countries/regions.
